# Better understanding care transitions of adults with complex health and social care needs: a study protocol

**DOI:** 10.1186/s12913-022-07588-0

**Published:** 2022-02-15

**Authors:** Catherine Hudon, Kris Aubrey-Bassler, Maud-Christine Chouinard, Shelley Doucet, Marie-France Dubois, Marlène Karam, Alison Luke, Grégory Moullec, Pierre Pluye, Amanda Tzenov, Sarah Ouadfel, Mireille Lambert, Émilie Angrignon-Girouard, Charlotte Schwarz, Dana Howse, Krystal Kehoe MacLeod, André Gaudreau, Véronique Sabourin

**Affiliations:** 1 Département de Médecine de Famille et Médecine d’urgence, Université de Sherbrooke (UdeS), Pavillon Z7-local 3007, 3001, 12e Avenue Nord, Sherbrooke, QC J1H 5N4 Canada; 2 Primary Healthcare Research Unit, Memorial University of Newfoundland (MUN), St-John’s, NL Canada; 3 Faculté des sciences infirmières, Université de Montréal (UdeM), Montreal, QC Canada; 4 Department of Nursing and Health Sciences, University of New-Brunswick (UNB), Fredericton, NB Canada; 5 Département des sciences de la santé communautaire, UdeS, Sherbrooke, QC Canada; 6 École de santé publique, Département de médecine sociale et préventive, UdeM, Montreal, QC Canada; 7 Department of Family Medicine, McGill University, Montreal, QC Canada; 8 Department of Family Medicine, MUN, St-John’s, NL Canada; 9 Centre intégré universitaire de santé et de services sociaux du Saguenay-Lac-Saint-Jean, Chicoutimi, QC Canada; 10 Postdoctoral Fellow, Department of Nursing and Health Sciences, UNB, Fredericton, NB Canada; 11 patient partner, Montreal, QC Canada

**Keywords:** Primary care, Frequent users, Chronic diseases, Care transition, Multi-site study

## Abstract

**Background:**

Adults with chronic conditions who also suffer from mental health comorbidities and/or social vulnerability require services from many providers across different sectors. They may have complex health and social care needs and experience poorer health indicators and high mortality rates while generating considerable costs to the health and social services system. In response, the literature has stressed the need for a collaborative approach amongst providers to facilitate the care transition process. A better understanding of care transitions is the next step towards the improvement of integrated care models. The aim of the study is to better understand care transitions of adults with complex health and social care needs across community, primary care, and hospital settings, combining the experiences of patients and their families, providers, and health managers.

**Methods/design:**

We will conduct a two-phase mixed methods multiple case study (quantitative and qualitative). We will work with six cases in three Canadian provinces, each case being the actual care transitions across community, primary care, and hospital settings. Adult patients with complex needs will be identified by having visited the emergency department at least three times over the previous 12 months. To ensure they have complex needs, they will be invited to complete INTERMED Self-Assessment and invited to enroll if positive. For the quantitative phase, data will be obtained through questionnaires and multi-level regression analyses will be conducted. For the qualitative phase, semi-structured interviews and focus groups will be conducted with patients, family members, care providers, and managers, and thematic analysis will be performed. Quantitative and qualitative results will be compared and then merged.

**Discussion:**

This study is one of the first to examine care transitions of adults with complex needs by adopting a comprehensive vision of care transitions and bringing together the experiences of patients and family members, providers, and health managers. By using an integrated knowledge translation approach with key knowledge users, the study’s findings have the potential to inform the optimization of integrated care, to positively impact the health of adults with complex needs, and reduce the economic burden to the health and social care systems.

**Supplementary Information:**

The online version contains supplementary material available at 10.1186/s12913-022-07588-0.

## Background

Adults with multiple chronic conditions who also suffer from mental health comorbidities and/or social vulnerability require services from many providers across different sectors, including the health and social care sectors. They have health and/or social care needs that are more complex than adults who need services from only one sector [1] and suffer from poorer health indicators and higher mortality rates while generating considerable cost to the health and social services system [2, 3]. In developed countries, nearly 70% of healthcare costs [4] are attributable to 10% of the population, mainly because of complex needs [5, 6].

Services organization for these adults with complex needs requires integrated care across providers of all settings and sectors [7, 8]. The World Health Organization proposes a user-led definition of integrated care to support strategies at all levels of the system: “*My care is planned with people who work together to understand me and my carer(s), put me in control, coordinate and deliver services to achieve my best outcomes*” [8, 9]. A recent systematic review of the effects of integrated care indicated improvement in quality of care, patient satisfaction, and access to care [10]. A meta-analysis investigating the impacts of integrated care showed a significant reduction of 19% in the probability of hospitalization when compared with usual care [11]. Models of integrated care, such as case management or the patient-centered medical home [8], aim to overcome fragmentation and improve care transitions as patients move across different settings and sectors [12]. Better understanding of care transitions of adults with complex needs is necessary mandatory to improve and implement these models of integrated care [13].

The Canadian Institutes of Health Research (CIHR) defines care transition as a transfer of responsibility between health and social care providers across different settings and sectors [14]. The patient’s journey through the system goes beyond only post-hospital care transitions and can involve a number of interfaces between home, community, primary care, and hospital settings [15], creating many points of transitions and patient-provider interactions, which are an integral part of a patient’s journey through the health and social care system [16]. In these care transitions, patients with complex needs often have to repeat their story, develop trust and new relationships with many providers, and make many important decisions. Problems in care transitions can seriously impact the four dimensions of the health and social care system performance [17] by: 1) affecting patients’ experience of their journey; 2) producing emotional and physical pain and suffering for patients and families, delaying appropriate treatment or support, increasing morbidity and mortality; 3) resulting in provider dissatisfaction with care coordination; and 4) leading to additional tests, primary care or emergency department visits, readmissions to hospital, producing considerable undue costs [15, 18].

Focusing on the care transitions of patients with complex needs is a priority [15] for health researchers, and a better understanding of these transitions, taking into account patients’ experiences is the next necessary step towards improvement and implementation of integrated care models [19]. Considerable evidence demonstrates that the experience of individuals and their families should be the central focus [20, 21] of efforts to better understand care transitions. Coleman et al. [13, 22] completed many studies in the United States of America (USA) to better understand challenges and opportunities for improving care transitions for older people, especially post-hospital care transitions. They developed the Care Transition Measure to provide insight into the quality of care transitions for post-hospital care [23], sought family caregivers’ experience regarding the challenges they faced facilitating their loved ones’ transitions [24], and evaluated an intervention for preparing older patients and caregivers to participate in post-hospital care transitions [25, 26]. Coleman et al. concluded that supporting patients and caregivers to take a more active role during care transitions appears promising for reducing rates of subsequent hospitalization. This evidence has led to the development of “toolkits” for guiding care transitions after hospital or emergency discharge, like the BestPATH evidence informed improvement package produced by Health Quality Ontario [27]. Coleman et al. [25], recommended that future studies should include more diverse populations. In Canada, Giosa et al. [28] and Backman et al. [29] examined the experiences of older adults and their families during care transitions between hospital and home [28] or across healthcare settings [29]. Their results stressed the need for active involvement of older adults and their families in managing care transitions [27].

In a study on patients’ experience transitioning between primary care and the emergency department in Belgium, Karam et al. [30] showed that patients with comorbidities perceived poor coordination between both levels of care. Other authors have also reported on the experience of providers with care transitions [31, 32]. In the USA, Davis et al. [31] reported that poor care transitions limited the ability of healthcare providers to provide optimal patient care. Lack of standardized processes, poor multidisciplinary communication, and fragmented communication across settings led to chaotic and challenging transitions; poor patient outcomes; and feelings of futility and dissatisfaction among providers. Providers reported that patients with complex needs were especially vulnerable during care transitions [31]. In Canada, Jeffs et al. [32] stressed the importance of having a collaborative approach amongst providers during the care transition process.

These previous studies and initiatives emphasize the important challenges faced by patients, their families, as well as care providers regarding care transitions. They also outline the importance of involving patients and their families in the care transitions, and the fact that adults with complex needs are particularly vulnerable to fragmented care. However, the experiences of non-geriatric patients remain understudied. Moreover, many studies only concern post-hospital discharge. While this transition appears important from a provider or manager perspective because of the risk of readmission and additional costs, the patient’s journey is much more complex and includes many transitions in care that are embedded in particular life contexts. We do not know much about patients’ experience of their whole journey, including transitions with organisations anchored in the patient’s neighborhood, such as community-based organisations.

### Research objectives

The objectives of the study are to: 1) identify individual and environmental characteristics of patients with complex needs that are associated with good or poor experiences of care transitions; 2) better understand the care transition experience of patients with complex needs and their families across community, primary care, and hospital settings; 3) better understand the experience of providers and health managers regarding care transitions of patients with complex needs; 4) examine care transitions by bringing together the experiences of patients and family members, providers, and community partners, as well as health managers.

## Methods/design

### Study design

The project will be grounded in the pragmatism paradigm which relies on the assumption that the finality of knowledge is to address concrete problems and provide answers or direction to progress [33]. We will conduct a two-phase mixed methods multiple case study (sequential explanatory design) [34, 35], starting with a quantitative phase (phase 1) to answer objective 1, followed by a qualitative phase (phase 2) for objectives 2 and 3. This design is well suited to answer research questions addressing complex systems in varied and dynamic contexts, allowing an in-depth analysis of each case, and offering opportunities for comparison between cases (objective 4) [36]. We will work with six cases in three provinces in Canada (Quebec, New Brunswick, and Newfoundland and Labrador), each case being the actual care transitions across community, primary care and hospital settings [37]. Each case will be called a health network. According to the conceptual model of factors affecting care transitions*,* presented thereafter [38], three levels of analysis will allow an in-depth understanding of each case: 1) the patient level; 2) the provider level; and 3) the healthcare system level.

Researcher and knowledge user collaboration throughout the research process is a strong predictor that research findings will be used [39]. In addition to traditional knowledge translation (KT) at the end of the project, we will carry out integrated KT by engaging knowledge users from each audience (patient partner, providers, decision-makers/managers) in the study’s Steering Committee. They will participate in key decisions throughout the study to ensure findings are useful to them in their respective contexts.

### Conceptual model

We used the conceptual model of factors affecting care transitions [38] to identify relevant independent variables to measure with questionnaires in phase 1, and to develop interview guides in phase 2. We chose this model because of its three-level structure of factors affecting care transitions and the relevance of the factors at each level: 1) the patient level (severity of illness, factors of vulnerability, self-management ability, social support); 2) the provider level (accountability, clarification of roles); and 3) the healthcare system level (access, coordination).

### Phase 1 (objective 1)

#### Sampling of the cases

Each case will be the actual care transitions across community, primary care, and hospital settings in each of the six health networks. To identify the cases, we targeted six emergency departments (ED), two per province, which are already engaged to participate in the study. The EDs were identified using a purposeful sampling strategy [40], to represent real-world differences [41] in terms of provinces, geographic area (rural, semi-urban, and urban), and both official languages (French and English). The inclusion of multiple cases capitalizes on organizational variation in care transitions to develop a more informed understanding. It also allows for observation of similar or singular care transitions, and draws conclusions that could be transferable to other contexts [42]. It is recommended that four to ten cases be considered [43] in the multiple case study logic of theoretical replication [42].

#### Sampling of patients with complex needs

Identifying patients with complex needs is a challenge because it does not depend on a precise diagnosis. We know that patients with complex needs frequently use many health and social services, and that ED visits are a good proxy of this use [44]. For screening, we will thus use our COmplex NEeds Case-finding Tool – 6 (CONECT-6) [45]. We validated this 6-question tool among patients at their third or more visit to the ED within 12 months, to screen those with complex needs (INTERMED Self-Assessment positive), with a sensitivity of 90% and a specificity of 66% with a threshold of two or more positive answers [45]. Patients screened as having complex needs with CONECT-6 will be invited to confirm their complex needs with INTERMED Self-Assessment (IMSA) [46]. IMSA is a self-reported version of the INTERMED questionnaire, taking 15 min to complete and measuring the complexity of adult needs. The first version of INTERMED was developed in the 1990s by an international team that combined their research expertise on complexity. Its psychometric qualities are well documented [47–51]. IMSA includes 20 questions subdivided into four domains: biological, psychological, social, and health system. Every domain is divided into three segments: history, current state, and prognosis. French and English-language versions are available. Patients with a score of 19 or higher will be invited to participate in the study, since this threshold confirms complex needs [52].

During the first year of the study, research assistants will be present in each ED four days a week to identify adults (≥ 18 years) at their third or more visit to the ED within 12 months, using the information system of the ED. Relying on a previous study [45], we estimate this number of patients at six per day in each ED (more in bigger EDs and fewer less in smaller EDs). Research assistants will invite those patients to answer CONECT-6 (two minutes). Approximately 30% of these patients will score positive on CONECT-6 [45] (*n* = 2 per day in each ED), and the research assistants will administer the IMSA questionnaire to those patients. CONECT-6 has a positive predictive value of 50% [45]; therefore, one patient will score complex on IMSA per day in each ED. Estimating an acceptance rate of 50%, we will recruit two patients per week for the project in each ED. We estimate a percentage of 60% of women and 40% of men [45]. It will then take about six months to recruit 180 women and nine months to recruit 180 men.

#### Data collection

At baseline, questionnaires with good psychometric properties in English and French will be administered by the research assistants to all participants, preferably during waiting at the ED, or by telephone (without affecting validity) [53] within two weeks of their ED visit. The questionnaires will collect information on age, sex, gender, indigenous identity, ethnicity, language, marital status, education, occupation, income, housing conditions, residential address, food security, social support, health literacy, alcohol, and drug use, multimorbidity, and self-management. These variables were identified based on the conceptual model [39]. Required time to complete the questionnaires is about 30 min. Age, language, marital status, education, occupation and income will be measured with questions from the Canadian Community Health Survey (CCHS) [54], sex and gender with the Statistics Canada census questions [55], ethnicity and indigenous identity with the Tri-Agency self-identification Equity and Diversity Questionnaire [56] (2 items), housing conditions with the Housing Satisfaction Question [57] (1 item), food security with the U.S. Household Food Security Survey Short Form [58] (6 items), social support with the Medical Outcomes Study Social Support Survey [59] (8 items), health literacy with the Brief Health Literacy Screening (BHLS) questionnaire [60, 61] (3 items), alcohol and drug use with the Alcohol, Smoking and Substance Involvement Screening Test (ASSIST) questionnaire [62] (9 items), multimorbidity with the Disease Burden Morbidity Assessment (DBMA) [63, 64] (21 items), and self-management with the Partners in Health Scale (PIH) [65, 66] (12 items). Environmental data such as neighborhood deprivation, gentrification, and marginalization will come from the Canadian Urban Environmental Health Research Consortium (CANUE) [67], using patients’ 6-digit postal codes.

At six months, we will administer a questionnaire by telephone to measure the experience of care transitions in the previous six months taking into consideration the patient’s holistic experience. The Patient Experience of Integrated Care Scale (12 items) [68], which we developed and validated from a set of items proposed by the Picker Institute Europe and the University of Oxford [69], will allow us to focus on the global experience of care transitions. The 12 items will result in a continuous score ranging from 0 to 48 where a higher score indicates better care integration.

#### Data analysis

Descriptive statistical analyses will be performed. The association between baseline independent variables and patient experience of care transitions (dependent variable measured at six months) will be assessed with multilevel linear regression models using SPSS V.24. Bivariate analyses (separate for each sex) will first be conducted using a cut-off level of α = 0.10 for inclusion in the multivariable model. The multilevel regression models (one for each sex) will then be reduced with backward elimination (α = 0.05) [70]. For all analyses, allowing for random intercepts and slopes with multilevel modeling will allow us to take into account the possible clustering of care transitions outcomes within the same health network. We estimate the intra-cluster correlation to be around 0.01, which is the median intra-cluster correlations estimated from Adams et al. [71] who examined 1039 variables from 31 studies in primary care. For each model, at the 5% significance level, a sample size of around 117 has 80% power for an expected medium effect size (R^2^ = 0,15) [72] with up to 10 predictors in the final multivariable models (G*Power 3.1.9.4) [73]. We find that 144 participants clustered within six cases are statistically equivalent to 117 independent participants, considering the design effect of (1 + [nm-1])*0.01) [74] where *n* = 24 women (or men) per case (144/6). We therefore need 288 patients (144 women and 144 men) completing the questionnaire at 6 months. Estimating a loss to follow-up of 20% [75, 76], 360 patients (180 women and 180 men) will be recruited at baseline in the EDs. For data security and privacy, all data will be hosted on hospital-grade internal servers. All data will be stripped of personally identifying information, and only the principal investigators from each province will have access to the key to identify individual participants within their province.

### Phase 2 (objectives 2 and 3)

#### Data collection

In person or virtual individual semi-structured interviews will be conducted to capture the richness of the perspectives [77, 78] with eight patients and family members per case (total *n* = 48), with a diversity of gender using a purposive sampling [79] among participants with the lowest and highest results for care transitions from phase 1. After providing written informed consent, each participant will do a one-hour interview conducted by a research assistant trained in qualitative research methods with a semi-structured interview guide composed of open-ended questions on their experience of care transitions. The interview guide developed for the interview with the patients and family members is provided as Additional file 1. The interviewer will take time to clearly explain the concept of care transitions with examples at the beginning. A few examples of questions are: What is working well in your care transitions (explain more if required) and why? Can you provide an example of a transition you felt good about and why? What is the most difficult in these transitions and why? Can you provide specific examples of transitions that were more challenging? How do you think the healthcare system could improve the way you experience these transitions? The research team’s patient partners will help refine and test the interview guides. The interviews will be digitally recorded and transcribed verbatim. We will aim for data saturation while expecting a certain variability among the cases [80], so the number of participants will be adjusted iteratively. About 40 interviews are usually needed to reach saturation in multisite studies [81].

In person or virtual focus groups (FG) of six providers (good balance between women and men) will be conducted in each case: one FG of family physicians, nurses’ practitioners, and specialists; one FG of other professionals including social workers; and one FG of community pharmacists and community organization partners. Key informants [82] and a snowball technique [83] will be used to identify providers who could share their experience to better understand care transitions of this population. Results of phase 1 will be presented to all FG participants to contextualize the discussion that will be facilitated with a semi-structured interview guide composed of open-ended questions on their experience of care transitions with this population. The interview guide developed for the focus group with the providers is supplied as Additional file 2. A few examples of questions are: What is going well, what is more difficult, and what should be done to improve these transitions? The FG will be digitally recorded and transcribed verbatim. We estimated the number of groups (3 groups per case) to reach data saturation [80, 81] for each category of providers. The optimal number of groups will be determined iteratively depending on the variability among the cases.

To explore the healthcare system level of our conceptual model, in person or virtual individual semi-structured interviews will be conducted with eight (good balance between women and men) health managers per case (total *n* = 48), working in different settings (hospitals, primary care, etc.), identified with key informants [82] and a snowball technique [83]. Results of phase 1 will be presented to health managers to contextualize the discussion that will be facilitated with an interview guide composed of open-ended questions on their experience. The interview guide developed for the interview with the managers is provided as Additional file 3. A few examples of questions are: What is going well, what is difficult, and what should be done at your managerial level to improve these transitions? The FG will be digitally recorded and transcribed verbatim. We estimated the number of interviews (eight per case) to reach data saturation [80, 81]. The optimal number of groups will be determined iteratively depending on the variability among the cases.

#### Data analysis

Two team members from different professional backgrounds will read the transcripts and iteratively analyze them using a deductive (themes based on the conceptual model of factors affecting care transitions) [38] and inductive (themes emerging from the data) thematic analyses [84]. Qualitative data will be managed using NVivo V.12 server software (QSR International Pty). To ensure credibility and minimize the effect of researcher subjectivity, the two team members will share and discuss results of the analysis with the team to confirm and enrich the findings. We will encourage pair debriefing and triangulation of researcher backgrounds and of collaborators’ expertise [85]. Transparency in analysis and reporting will be achieved by providing extensive deidentified verbatim quotes and a detailed description of the contexts, which will also help promote transferability to similar contexts.

### Integration of quantitative and qualitative results (objective 4)

Two types of integration will be performed [86]. First, qualitative and quantitative results will be compared. Then for each case, qualitative and quantitative data will be merged [42]. A case summary will be reported (synthesizing merged data), and the six case summaries will be used to compare cases by means of a descriptive and interpretative matrix, allowing systematic comparisons among cases and analysis units (patient, provider, and health manager levels). Different analytical techniques will be used such as pattern comparison, research of competing explanations, and construction of explanations [42]. Management, reduction, and comparisons will be conducted with NVivo V.12 software. Knowledge users on the steering committee of this project will participate in key steps of the analysis to ensure meaningful interpretation [87, 88]. Deidentified case summaries could be used as “vignettes” in the knowledge translation plan and the web interactive learning module to illustrate good or poor care transitions. Knowledge users, including patient partners, will be involved in the knowledge translation plan.

## Discussion

This project is innovative and creative in many ways. It will be one of the few studies examining care transitions of adults with complex needs and adopting a comprehensive vision of care transitions. In line with the pan-Canadian Strategy for Patient-Oriented Research (SPOR) [87], we will focus on patients’ lived experiences of care transitions. We propose to bring together the experiences of patients and family members, providers, and community partners such as health managers.

The results of this project will be of interest to researchers as well as to decision-makers in the healthcare system. Our understanding of the patients’ experience of their whole journey regarding care transitions is very limited. Through this project, we expect to contribute new knowledge about the care transitions of adults with complex needs using a comprehensive vision of care transitions. This project will help inform decision-makers, specifically regarding how individual and environmental characteristics of patients with complex needs are associated with good or poor experiences of care transitions. Many strategies will thus be undertaken during the multiple case study, so that project conclusions can be transferable to other contexts: theoretical enlightenment; reproduction of observations in many cases; and in-depth description of context, facilitators, and barriers in care transitions.

Many patients with complex needs often use health and social services. However, few patients with complex needs are not frequent ED users, so these patients will be missed by our inclusion criteria focusing on ED visits. Also, results could be transferred to networks presenting similar characteristics. Recruitment of six different networks and a detailed description of their context will promote transferability.

## Supplementary Information

**Additional file 1.** Interview guide for the interview with the patients and family members.

**Additional file 2.** Interview guide for the focus group with the providers.

**Additional file 3.** Interview guide for the interviews with the managers.

## Data Availability

A de-identified dataset/transcripts may be made available upon reasonable request of the corresponding author once the study is complete.

## Abbreviations

ASSIST: Alcohol, Smoking and Substance Involvement Screening Test
BHLS: Brief Health Literacy Screening
CANUE: Canadian Urban Environmental Health Research Consortium
CCHS: Canadian Community Health Survey
CIHR: Canadian Institutes of Health Research
CONECT-6: Complex Needs Case-finding Tool-6
DBMA: Disease Burden Morbidity Assessment
ED: Emergency Department
FG: Focus groups
IMSA: INTERMED Self-Assessment positive
USA: United States of America
PROMs: Patient Oriented Outcome Measures
PIH: Partners in Health Scale
SPOR: Canada’s Strategy for Patient-Oriented Research strategy
