# Supplementary material for: Better understanding care transitions of adults with complex health and social care needs: a study protocol
Source: BMC Health Serv Res. 2022 Feb 15;22:206. doi: 10.1186/s12913-022-07588-0 (PMC8848684; doi:10.1186/s12913-022-07588-0)
Supplement: Supplementary file 1 — Additional file 1. Interview guide for the interview with the patients and family members. [file 12913_2022_7588_MOESM1_ESM.pdf]

Additional file 1. Interview guide for the interviews with the patients and family members
To be shared with the participant before the interview

Reference Number: ______________________________ Date: ____________________________________

Location: ____ Time: ________________________

Interviewer: ____

INTRODUCTION

Hello, my name is ____________. I am a researcher for this study called *Better understanding care transitions of adults with complex health and social care needs: a study protocol*. I would like to thank you for agreeing to participate in this interview.

We have invited you to be interviewed as part of this study because you have personal experience of receiving care or services from different healthcare providers and settings and we would like to hear your thoughts about your journey in the healthcare system. Our team is looking to understand your experience about your 'care transitions'. These care transitions refer to those times when you move between different healthcare settings and healthcare providers, for example, from home to emergency room, from a medical clinic to your community pharmacy or services offered by a community organization, as your conditions and care needs change. It can be for a new service from a healthcare provider or organization or, after having received new services, when you return to see a provider you already know. Each of these shifts between care providers and settings is considered a care transition. Before starting, I want to tell you that there is no wrong answer. The best answer will be what you think when you first hear the questions. So, please, feel free to express yourself and ask questions if anything is unclear.

First, I would like to walk you through the consent form. Please let me know if you have any questions

[FOR FACILITATOR: REVIEW INFORMED CONSENT FORM AND ANSWER ANY QUESTIONS.]

Before we begin our discussion, I would like to spend a few moments talking our discussion today.

- We may use quotes collected during our discussion today, but no names will be associated with the quotes and you would not be able to be identified.
- If you feel uncomfortable at any time or if you would like to take a break, just let me know
- I would like to remind you that this interview will be audio recorded to increase accuracy and to reduce the chance of misinterpreting your comments.
- Audio recordings and their transcripts will be password protected. Only the research team will have access to this data. The audio recordings will be deleted as soon as they have been transcribed.
- Your name will be removed from the transcript and labelled with a study code that will be linked to your name in a separate document. Only the research team will have access to this study code document.
- I will be taking notes during the course of our discussion today.

Unless you have any questions, we can begin. I am now turning on the audio recording device.

[START THE AUDIO RECORDING DEVICE]

INTERVIEW QUESTIONS

1. Please talk to me about your health. *Probes: health problems (which ones, for how long), particular challenges related to, impacts (functional, emotional, social), coping, self-management ability.*

2. Could you describe your day-to-day life? *Probes: context of life, socio-economic conditions, social support, vulnerability factors (food security, housing, etc.).*

3. Please describe your interactions with the healthcare system and different community health or social care organizations in the last six months. *Probes: enrolment to a primary care provider, emergency room visits, hospitalizations, follow-up by specialists, visits with other healthcare or social services professionals, use of community organizations' services.*

4. *We will use answers from #3 to frame the question. For example,* "You mention a couple of visits to the emergency room, can you describe that transition following those visits back to your primary care provider? OR you said you were referred to a specialist last year, can you describe what that was like?" *Probes: access, collaboration and communication among providers, consideration of their needs, involvement in decision-making.*

5. What is working well in these transitions (explain more if required) and why? Provide transition examples that are working well. *Probes: factors related to the healthcare system level (access, coordination); factors related to the provider level (collaboration and communication among providers, consideration of their needs, involvement in decision-making); factors related to the patient level (severity of illness, factors of vulnerability, self-management ability, social support).*

6. What is more difficult in these transitions and why? Provide specific examples of transitions that are more challenging. *Probes: factors related to the healthcare system level (access, coordination); factors related to the provider level (collaboration and communication among providers, consideration of their needs, involvement in decision-making); factors related to the patient level (severity of illness, factors of vulnerability, self-management ability, social support).*

7. How do you think we could improve the way you experience these transitions? Provide specific examples on how transitions can be improved. *Probes: access, collaboration and communication among providers, consideration of their needs, involvement in decision-making.*

8. Is there anything else you would like to share with us about transitions in care?
