# Supplementary material for: Better understanding care transitions of adults with complex health and social care needs: a study protocol
Source: BMC Health Serv Res. 2022 Feb 15;22:206. doi: 10.1186/s12913-022-07588-0 (PMC8848684; doi:10.1186/s12913-022-07588-0)
Supplement: Supplementary file 2 — Additional file 2. Interview guide for the focus group with the providers. [file 12913_2022_7588_MOESM2_ESM.pdf]

Additional file 2. Interview guide for the focus groups with the providers
To be shared with the participant before the interview

Reference Number: ______________________________ Date: _____________________________________

Location: ____ Time: _______________________

Interviewer: ____

INTRODUCTION

Hello, my name is ____________. I am a researcher for this study called *Better understanding care transitions of adults with complex health and social care needs: a study protocol*. I would like to thank you for agreeing to participate in this focus group.

We have invited you to be interviewed as part of this study because we are interested in your experiences as a healthcare provider. Specifically, we would like to hear about your experiences about transitions in care for patients with complex needs across community, primary care and hospital settings. Before starting, I want to tell you that there are no wrong answers. The best answer will be what you think when you first hear the question. So, please, feel free to express yourself and ask questions if anything is unclear.

In a few minutes, we will all introduce ourselves. You may use your first name or a made-up name if you prefer. But first, I would like to walk you through the consent form. Please let me know if you have any questions

[FOR FACILITATOR: REVIEW INFORMED CONSENT FORM AND ANSWER ANY QUESTIONS.]

Before we begin our discussion, I would like to spend a few moments talking about confidentiality and basic ground rules for our focus group discussion today.

- Everyone's views will be welcomed.
- We may use quotes collected during our discussion today, but no names will be associated with the quotes and you would not be able to be identified.
- We encourage everyone to respect the views of others, and therefore ask that you keep the opinions you heard today confidential. Having said this, we cannot guarantee that everyone in the room will honour this request.
- Therefore, we ask that you make only comments you would feel comfortable making in a public setting.
- I will do my best to ensure that everyone has a chance to speak.
- I may also step in if the conversation is straying off topic.
- If you feel uncomfortable at any time or if you would like to step out, a member of our team can go with you and make sure you are OK.

I would like to remind you that this focus group will be audio recorded to increase accuracy and to reduce the chance of misinterpreting anyone's comments.

- Audio recordings and their transcripts will be password protected. Only the research team will have access to this data. The audio recordings will be deleted as soon as they have been transcribed.
- Participants' names will be removed from the transcripts and labelled with study codes that will be linked to participants' names in a separate document. Only the research team will have access to this study code document.
- For transcription purposes, I would ask that for the first few comments, you say your first name so that I will recognize your voice. I may give you a gentle reminder to do so.
- I will be taking observer notes during the course of our discussion today.

Unless you have any questions, we can begin. I am now turning on the audio recording device.

I will begin by presenting you results of the first phase of our study about patients' characteristics associated with good or poor care transitions and then I will ask you questions about care transitions.

[FOR FACILITATOR: REVIEW PHASE 1 STUDY FINDINGS.]

[START THE AUDIO RECORDING DEVICE]

I would like to invite you to first introduce yourself to one another using your first name or a made-up name.

FOCUS GROUP QUESTIONS

1. Please tell me about your role as a care provider in the healthcare system. *Probes: role, since when, where.*

2. Please tell me about your experience with patients with complex needs regarding their transitions between home, emergency room, hospital, consultations with different healthcare providers, other individuals associated with care, and community organizations. *Probes: access, collaboration and communication among providers, clarification of providers' roles, consideration of their needs, involvement in decision-making.*

3. What is working well in these transitions and why? Provide transition examples that are working well. *Probes: factors related to the healthcare system level (access, coordination); factors related to the provider level (collaboration and communication among providers, consideration of their needs, involvement in decision-making); factors related to the patient level (severity of illness, factors of vulnerability, self-management ability, social support).*

4. What is more challenging with these transitions and why? Provide specific examples on transitions that are more challenging. *Probes: factors related to the healthcare system level (access, coordination); factors related to the provider level (collaboration and communication among providers, consideration of their needs, involvement in decision-making); factors related to the patient level (severity of illness, factors of vulnerability, self-management ability, social support).*

5. How do you think we can improve these transitions? Provide specific examples on how transitions can be improved. *Probes: access, collaboration and communication among providers, consideration of their needs, involvement in decision-making.*

6. Is there anything else you would like to share with us about transitions in care?

Wrap up (2 minutes)

That concludes our discussion for today. I would like to remind you that what has been said here today should remain confidential. **[THANK PARTICIPANTS]**
